# Comparing baseline and longitudinal measures in association studies

**DOI:** 10.1186/1753-6561-8-S1-S84

**Published:** 2014-06-17

**Authors:** Shuai Wang, Wei Gao, Julius Ngwa, Catherine Allard, Ching-Ti Liu, L Adrienne Cupples

**Affiliations:** 1 Department of Biostatistics, Boston University School of Public Health, 801 Massachusetts Avenue 3rd floor, Boston, MA 02118, USA; 2 Département de Mathématiques, Université de Sherbrooke, Québec, Canada J1K 2R1

## Abstract

In recent years, longitudinal family-based studies have had success in identifying genetic variants that influence complex traits in genome-wide association studies. In this paper, we suggest that longitudinal analyses may contain valuable information that can enable identification of additional associations compared to baseline analyses. Using Genetic Analysis Workshop 18 data, consisting of whole genome sequence data in a pedigree-based sample, we compared 3 methods for the genetic analysis of longitudinal data to an analysis that used baseline data only. These longitudinal methods were (a) longitudinal mixed-effects model; (b) analysis of the mean trait over time; and (c) a 2-stage analysis, with estimation of a random intercept in the first stage and regression of the random intercept on a single-nucleotide polymorphism at the second stage. All methods accounted for the familial correlation among subjects within a pedigree. The analyses considered common variants with minor allele frequency above 5% on chromosome 3. Analyses were performed without knowledge of the simulation model. The 3 longitudinal methods showed consistent results, which were generally different from those found by using only the baseline observation. The gene *CACNA2D3*, identified by both longitudinal and baseline approaches, had a stronger signal in the longitudinal analysis (*p *= 2.65 × 10^−7^) compared to that in the baseline analysis (*p *= 2.48 × 10^−5^). The effect size of the longitudinal mixed-effects model and mean trait were higher compared to the 2-stage approach. The longitudinal results provided stable results different from that using 1 observation at baseline and generally had lower *p *values.

## Background

Longitudinal data analyses are widely used in genome-wide association studies to assess genetic and environmental risk factors and their association with phenotypes of interest [1-3]. They are more complicated than analyses using only baseline measures because subjects are followed over time and change is measured during follow-up. Standard linear regression techniques are not applicable in this setting because of the correlation that exists among the repeated measures per subject. Methods for longitudinal study designs have enabled the investigation of genetic variation influencing trait values over time [3]. In Genetic Analysis Workshop 13, Gauderman et al [4] provided an overview of a wide range of methods for the genetic analysis of longitudinal data in families. They summarized these methods into 2 groups: (a) 2-stage approaches, in which a summary statistic is obtained and used in genetic analysis, and (b) joint modeling, in which the genetic and longitudinal data are analyzed simultaneously in a single analysis. They argued that the use of a mean-type statistic could provide greater power compared to a slope-type statistic for detecting a gene effect. Zhu et al [1] performed a genome-wide association in which they identified genes and gene-environment interactions associated with longitudinal traits. They implemented a multivariate adaptive spline for the analysis of the longitudinal data.

In this paper, our main object is to compare existing methods of longitudinal data analyses with those that use only 1 baseline measure in association studies. We explore the following longitudinal methods: (a) a longitudinal mixed-effects model; (b) analysis of the mean trait over time; and (c) a 2-stage analysis, with estimation of a random intercept in the first stage and regression of the random intercept on a single-nucleotide polymorphism (SNP) in the second stage. These longitudinal methods use statistics that capture the level of a trait, such as a mean, to detect genetic associations as opposed to methods that focus on the change in the trait over time, such as a slope. Despite the strengths and integrated approach of a longitudinal mixed model, its implementation is very computer-intensive because of its complex structure. Therefore, the main motivation for trying some "simpler" alternative longitudinal models, such as analysis of the mean trait over time and a 2-stage analysis, is to see if they can serve as good substitutes with equally good performance.

## Methods

### Study subjects and phenotype

We used real phenotype data collected in the San Antonio Family Heart Study, including sex, age, year of examination, systolic and diastolic blood pressure, use of antihypertensive medications, and tobacco smoking at up to 4 time points for 939 subjects in 20 pedigrees. Of the 939 participants, 244 attended only 1 exam; for the remaining subjects, the median follow-up time was 11 years with a median gap time between assessments of 5 years. We analyzed 2 continuous traits: systolic blood pressure (SBP) and diastolic blood pressure (DBP). For participants on medication, we imputed both SBP and DBP to mimic what their unmedicated values would be. If a subject was on medications at an exam, we imputed the blood pressure at this exam to be the average blood pressure of all observations with higher values among those of the same gender and ± 10 years of the age of the subject. We performed a preliminary analysis to select covariates for both SBP and DBP. Variables significantly associated (*p *<0.05) with SBP or DBP were selected. For SBP, we adjusted for age, sex, and tobacco smoking. For DBP, we adjusted for age, sex, tobacco smoking, and centered age squared.

### Genetic data

The genetic data from Genetic Analysis Workshop 18 (GAW18) consisted of whole genome sequence data in a pedigree-based sample with longitudinal phenotype data for hypertension and related traits. A total of 26.8 million SNPs were identified in the 483 individuals. After eliminating 19 outlier individuals who failed to meet SNP quality control criteria such as fractions and ratio of homogeneous and heterogeneous sites and fraction of novel SNPs, 24 million SNPs passed support vector machine and indel proximity filters. Genotype calls cleaned of mendelian errors and dosages were provided for 959 individuals (464 directly sequenced and the rest imputed) for 8,348,674 locations in the genome. A majority of the SNPs were rare variants; 51% had a minor allele frequency (MAF) below 1%. As suggested by GAW18 leaders, all analyses for this current paper were based on 402,985 common variants (MAF ≥5%) of chromosome 3 only, accounting for around one-third of the total number of variants on the chromosome.

### Statistical analyses

#### Baseline association analysis

For comparison with the methods that used the longitudinal data, we applied a baseline association analysis that considered only the first observation (baseline) for each person. In addition to adjusting for covariates, we incorporated a familial correlation structure (kinship coefficient matrix) into the model as $Y_{ij0}=\beta_{0}+X_{ij0}\;\beta +\beta_{s}SNP_{ij}+\alpha_{ij}+\varepsilon_{ij}$, where *i *denotes the *i*^th ^pedigree, and *j *denotes the *j*^th ^individual in the *i*^th ^pedigree. For this individual, $Y_{ij0}$ denotes the phenotype at baseline, $X_{ij0}=\left(X_{ij01},\ldots ,X_{ij0m}\right)$ denotes the covariates at baseline, and $SNP_{ij}$ denotes the SNP dosage. $\beta_{0}$ is the fixed intercept, $\beta ={\left(\beta_{1},\beta_{2},\ldots ,\beta_{m}\right)}^{\prime }$ is a vector of regression coefficients for the *m *covariates, and $\beta_{s}$ is the SNP effect size; $\alpha_{ij}$ is the random intercept for the (*i,j*)^th ^person. Within each pedigree, the $vector\;\alpha_{i}=\left(\alpha_{i1},\ldots ,\alpha_{in_{i}}\right)$ is normally distributed with a mean of 0 and a covariance matrix of $\sigma^{2}\Sigma_{kin}$ (the kinship matrix), contributing a diagonal block for each pedigree to the overall covariance matrix; $\varepsilon_{ij}$ is an error term with a mean of 0 and a variance of $\sigma_{\varepsilon }^{2}$. This model was implemented using the lmekin package in R (version 2.9.2) package "kinship" [5], which employed maximum likelihood methods to estimate parameters.

The notations of $\beta_{0},\beta ,\beta_{s},SNP_{ij},\alpha_{ij},\varepsilon_{ij}$ used in this baseline model apply to the following models where applicable.

To compare with the baseline approach, we considered 3 approaches for longitudinal analyses of these data: (a) longitudinal mixed-effects association analysis, (b) mean measure in longitudinal association analysis, and (c) 2-stage longitudinal association analysis.

#### Longitudinal mixed-effects association analysis

We used a random-intercept mixed effects model with familial correlation structure [7]. The model is:

(1) $$Y_{ijt}=\beta_{0}+X_{ijt}\beta +\beta_{s}SNP_{ij}+\alpha_{ij}+\varepsilon_{ij}$$

Here *i *denotes the *i*^th ^pedigree, and *j *denotes the *j*^th ^individual in the *i*^th ^pedigree. For this individual, $Y_{ijt}$ denotes the trait at time point t; $X_{ijt}=\left(X_{ijt1},X_{ijt2},\ldots ,X_{ijtm}\right)$ denotes the covariates at time t, including time-dependent covariates. This model was implemented in the R (version 2.15.1) package "pedigreemm" [6], which used the method of restricted maximum likelihood for parameter estimation.

#### Mean measure in longitudinal association analysis

We also considered the mean across all time points as the trait and its corresponding averaged covariates as one alternative for longitudinal association analysis. This model is:

(2) $$Y_{ij}^{*}=\beta_{0}+X_{ij}^{*}\beta +\beta_{s}SNP_{ij}+\alpha_{ij}+\varepsilon_{ij}$$

Here *i *denotes the *i*^th ^pedigree, and *j *denotes the *j*^th ^individual in the *i*^th ^pedigree. For this individual, $Y_{ij}^{*}$ denotes the mean trait across time. $X_{ij}^{*}=\left(X_{ij1}^{*},X_{ij2}^{*},\ldots ,X_{ijm}^{*}\right)$ denotes the covariates, which for time-dependent covariates is the average measure across time.

This model was implemented using the function lmekin in R (version 2.9.2) package "kinship" [5], using maximum likelihood methods to estimate parameters.

#### Two-stage longitudinal association analysis

Another longitudinal approach employs a 2-stage strategy [4]. In the first stage, a random intercept, $\alpha_{\text{ij}}$, as the level of the trait for each person was generated from a growth curve model:

(4) $${\text{Y}}_{\text{ijt}}=\beta_{10}+{\text{X}}_{\text{ijt}}\beta +\alpha_{\text{ij}}+\varepsilon_{\text{ij}}$$

Here *i *denotes the *i*^th ^pedigree, and *j *denotes the *j*^th ^individual in the *i*^th ^pedigree. For this individual, ${\text{Y}}_{\text{ijt}}$ denotes the trait at time point t. ${\text{X}}_{\text{ijt}}=\left({\text{X}}_{\text{ijt}1},{\text{X}}_{\text{ijt}2},\ldots ,{\text{X}}_{\text{ijtm}}\right)$ denotes the covariates including time-dependent covariates. $\beta_{10}$ is the fixed intercept of the first stage; $\alpha_{\text{ij}}$ is the random intercept. As above, the covariance structure of $\alpha_{\text{ij}}$ is $\sigma^{2}\Sigma_{\text{kin}},$ which contributes a diagonal block for each pedigree to the overall covariance matrix.

In the second stage, random intercept $\alpha_{\text{ij}}$ is treated as the "new" trait and regressed on a SNP as follows:

(5) $$\alpha_{ij}=\beta_{20}+\beta_{s}SNP_{ij}+\gamma_{ij}+\varepsilon_{ij}$$

Here $\text{SN}{\text{P}}_{\text{ij}}$ denotes the SNP dosage. $\beta_{20}$ is the intercept of the second stage; $\beta_{\text{s}}$ is the SNP effect size; $\varepsilon_{ij}$ is an error term with a mean of 0 and a variance of $\sigma_{\varepsilon }^{2},$$\gamma_{ij}$ is the random intercept that adjusts for the familiar correlation of $\alpha_{ij}$; and, similarly, the vector $\gamma_{i}=\left(\gamma_{i1},\ldots ,\gamma_{in_{i}}\right)$ is normally distributed with a mean of 0 and a covariance matrix of $\sigma_{\gamma }^{2}\Sigma_{kin},$ contributing a diagonal block for each pedigree to the overall covariance matrix.

Gauderman et al [4] pointed out that a mean-based statistic is more powerful to detect a genetic association than a slope-based statistic (eg, a random slope). So here we adopted the random intercept of the first stage rather than the random slope as the "trait" in the second stage. The first-stage model was implemented using lmekin of the R (version 2.15.1) package "coxme" [6], which could handle more than 1 random effect; the second-stage model was implemented using lmekin of the R (version 2.9.2) package "kinship"[5], which adopted a faster computing algorithm. Both packages used maximum likelihood in parameter estimation.

### Power and type I error

We conducted power calculations for all 4 methods and evaluated type I error by means of the genomic control value. We chose the variant (chromosome 3: 47956424) on gene *MAP4*, the top variant influencing simulated SBP and DBP, as the functional variant for power calculations. To determine power, we tested the null hypothesis that the trait SBP was not associated with the functional variant, versus the alternative hypothesis that it is associated. Therefore, results would be considered statistically significant if the *p *value obtained using the analysis methods fell below a predetermined threshold. Here we divided the significance level 0.05 by the approximate number (25,676) of independent SNPs on chromosome 3 to adjust for multiple testing. We used PLINK (http://pngu.mgh.harvard.edu/~purcell/plink/) [8] to prune out SNPs on chromosome 3 where the pairwise linkage disequilibrium was 0.2 or greater, and 25,676 SNPs remained. For each of the 4 methods, the estimated power was the proportion of replicates in which the method detected a significant association between the trait and the functional variant.

For each of the 4 methods, genomic control value was used to assess the extent of the inflation of type I error, based on the *p *value of common variants on chromosome 3.

## Results

### Association analysis of real data

**Figure 1:**
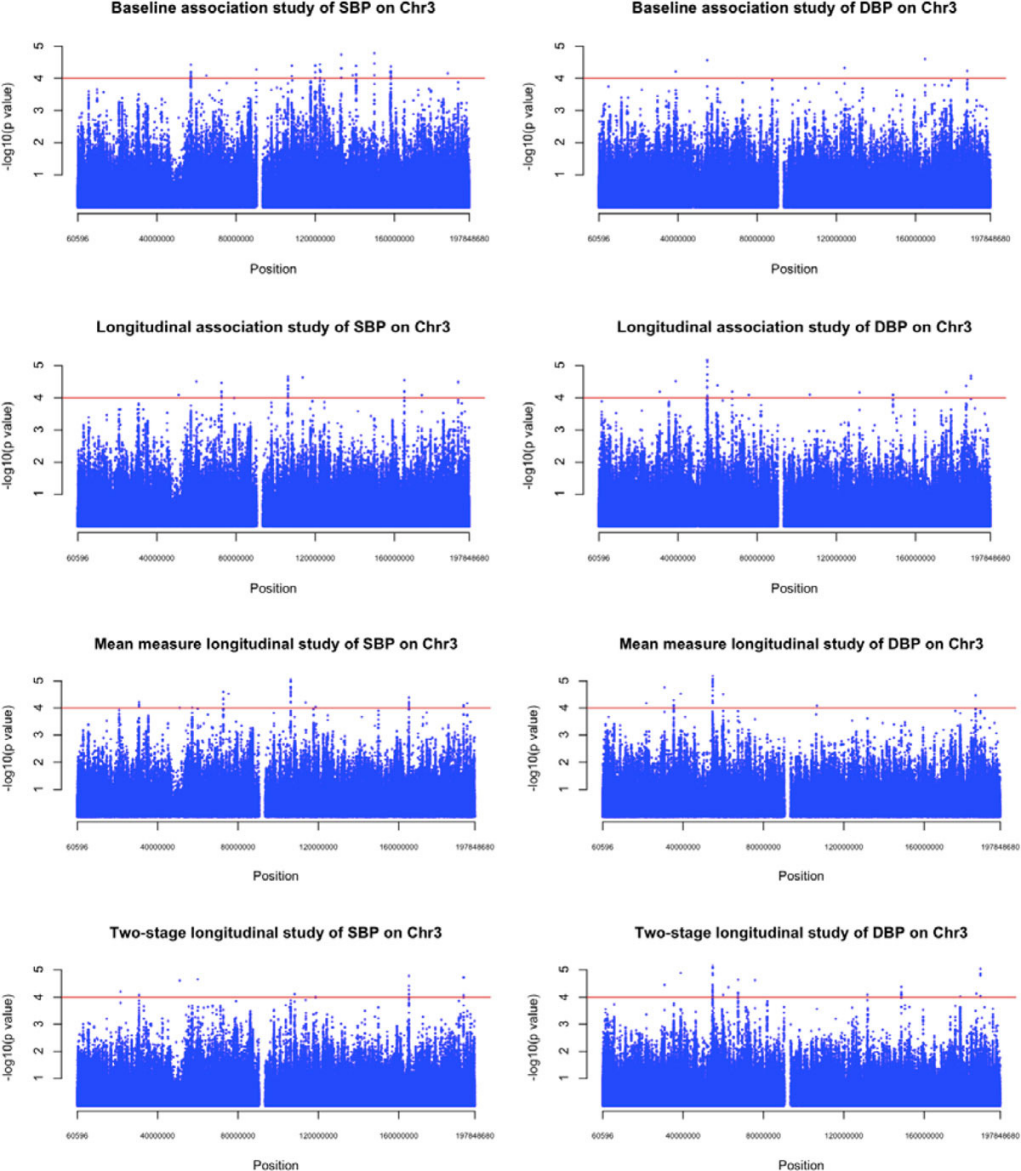
**Manhattan plots on chromosome 3 using both the baseline and 3 longitudinal methods for SBP and DBP**.

For SBP, there were no shared results in the top 10 hits between the baseline approach and the other 3 longitudinal methods (Table 1). Some shared genes identified by the longitudinal methods were *FGF12 *and *FHIT*. The mean measure and 2-stage methods yielded similar results. For DBP, the 3 longitudinal methods yielded consistent results (as shown in Figure 1, right side): the top 10 hits came from the same gene (*CACNA2D3 *in Table 2; eg, SNP 3_54748234 has a *p *value of 2.65 × 10^−7^), with SNPs nearly reaching a Bonferroni significance threshold. This gene was also found using the baseline method but was less significant (rank = 2, *p *= 2.76E-05 in Table 2).

**Table 1:** TOP 10 hits of SBP on chromosome 3 across the baseline method and the 3 longitudinal methods

Baseline					Longitudinal					Mean measure					Two-stage				
**SNP**	**Effect size**	**SE**	**P**	Closest* genes	**SNP**	**Effect size**	**SE**	**P**	Closest* genes	**SNP**	**Effect size**	**SE**	**P**	Closest* genes	**SNP**	**Effect size**	**SE**	**P**	Closest* genes

3_149871159	5.31	1.22	1.63E-05	*LOC646903*,	3_106220130	−4.36	1.02	2.16E-05	*LOC100302640*	3_106220130	−4.68	1.05	9.16E-06	*BCHE*	3_165046920	2.96	0.68	1.33E-05	*SLITRK3*

3_133160911	4.02	0.93	1.83E-05	***BFSP2***	3_113652027	3.77	0.89	2.34E-05	***GRAMD1C***	3_106220437	−4.65	1.05	1.06E-05	***FGF12***	3_59966975	2.52	0.58	1.59E-05	***FHIT***

3_149894219	5.10	1.22	3.41E-05	*LOC646903*,	3_106217172	−4.33	1.03	2.57E-05	*LOC100302640*	3_106217172	−4.66	1.05	1.07E-05	***FGF12***	3_192240010	−3.67	0.86	2.14E-05	***FGF12***

3_122390279	5.24	1.26	3.65E-05	*PARP14*	3_165046920	4.21	1.00	2.82E-05	*SLITRK3*	3_106218053	−4.62	1.07	1.64E-05	***FHIT***	3_192239815	−3.81	0.90	2.31E-05	***FGF12***

3_57173021	3.87	0.93	3.75E-05	***IL17RD***	3_106220437	−4.29	1.02	2.89E-05	*LOC100302640*	3_106219390	−4.62	1.07	1.64E-05	***DOCK3***	3_50996289	4.18	0.99	2.55E-05	***DOCK3***

3_120020489	3.73	0.90	3.95E-05	*LRRC58*	3_59966975	3.58	0.86	3.15E-05	***FHIT***	3_106231571	−4.54	1.05	1.71E-05	*BCHE*	3_165049274	2.75	0.66	3.35E-05	*SLITRK3*

3_120023242	3.73	0.90	3.95E-05	*LRRC58*	3_192239815	−5.47	1.31	3.20E-05	***FGF12***	3_106232849	−4.51	1.05	1.86E-05	*BCHE*	3_165046402	2.71	0.66	4.21E-05	*SLITRK3*

3_108188993	−3.77	0.91	4.00E-05	***MYH15***	3_192240010	−5.28	1.27	3.27E-05	***FGF12***	3_106220258	−4.39	1.02	1.86E-05	***ZNF385D***	3_165053404	2.63	0.66	6.83E-05	*SLITRK3*

3_140640076	7.59	1.84	4.08E-05	*SLC25A36*	3_72678387	−4.30	1.04	3.38E-05	*SHQ1*	3_106220368	−4.45	1.05	2.41E-05	***MYH15***	3_21520730	4.60	1.16	7.82E-05	***ZNF385D***

3_158228266	−4.87	1.18	4.31E-05	***RSRC1***	3_106218053	−4.28	1.04	4.20E-05	*LOC100302640*	3_72678387	−4.53	1.07	2.56E-05	*BCHE*	3_113652027	2.40	0.60	7.94E-05	***GRAMD1C***

*In the field "closest genes"; a bold gene name indicates that the SNP on the same row is right on that gene.

**Table 2:** TOP 10 hits of DBP on chromosome 3 across the baseline method and the 3 longitudinal methods

Baseline					Longitudinal					Mean-measure					Two-stage				
**SNP**	**Effect size**	**SE**	**P**	Closest* genes	**SNP**	**Effect size**	**SE**	**P**	Closest* genes	**SNP**	**Effect size**	**SE**	**P**	Closest* genes	**SNP**	**Effect size**	**SE**	**P**	Closest* genes

3_164797024	4.98	1.17	2.48E-05	***SI***	3_54748234	2.20	0.43	2.65E-07	***CACNA2D3***	3_54748234	2.23	0.44	3.73E-07	***CACNA2D3***	3_54757032	1.10	0.21	1.83E-07	***CACNA2D3***

3_54748368	−2.15	0.51	2.76E-05	***CACNA2D3***	3_54757032	2.21	0.43	3.21E-07	***CACNA2D3***	3_54757032	2.22	0.44	5.77E-07	***CACNA2D3***	3_54748234	1.11	0.21	2.39E-07	***CACNA2D3***

3_124142019	2.85	0.69	4.62E-05	***KALRN***	3_54748368	−2.15	0.42	3.84E-07	***CACNA2D3***	3_54748368	-2.17	0.43	6.80E-07	***CACNA2D3***	3_54793253	1.05	0.21	6.86E-07	***CACNA2D3***

3_186144694	−3.17	0.78	5.83E-05	*LOC253573*	3_54784952	2.11	0.43	7.90E-07	***CACNA2D3***	3_54793253	2.09	0.43	1.67E-06	***CACNA2D3***	3_54799449	1.05	0.21	7.79E-07	***CACNA2D3***

3_38845381	−3.68	0.91	6.06E-05	*SCN10A*	3_54793253	2.08	0.42	9.59E-07	***CACNA2D3***	3_54784952	2.09	0.44	2.05E-06	***CACNA2D3***	3_54784952	1.05	0.21	7.90E-07	***CACNA2D3***

3_186209848	−3.05	0.78	0.000107	*LOC253573*	3_54779240	2.08	0.43	1.04E-06	***CACNA2D3***	3_54799449	2.09	0.44	2.31E-06	***CACNA2D3***	3_54748368	−1.05	0.21	8.15E-07	***CACNA2D3***

3_87619500	2.32	0.60	0.000109	*POU1F1*	3_54756448	−2.10	0.43	1.07E-06	***CACNA2D3***	3_54756448	-2.09	0.44	2.35E-06	***CACNA2D3***	3_54779240	1.03	0.21	9.61E-07	***CACNA2D3***

3_177961323	−2.05	0.53	0.000115	*KCNMB2-IT1*	3_54756196	2.06	0.43	1.46E-06	***CACNA2D3***	3_54756196	2.08	0.44	2.48E-06	***CACNA2D3***	3_54807320	1.03	0.21	9.67E-07	***CACNA2D3***

3_72651668	2.05	0.53	0.000132	*SHQ1*	3_54793450	−2.07	0.43	1.57E-06	***CACNA2D3***	3_54747244	2.07	0.44	2.65E-06	***CACNA2D3***	3_54756196	1.03	0.21	1.26E-06	***CACNA2D3***

3_186149493	−3.04	0.79	0.000134	*LOC253573*	3_54740011	2.05	0.43	1.75E-06	***CACNA2D3***	3_54740011	2.07	0.44	2.67E-06	***CACNA2D3***	3_54799706	1.02	0.21	1.27E-06	***CACNA2D3***

*In the field "closest genes"; a bold gene name indicates that the SNP on the same row is right on that gene.

### Power and type I error

Power was computed to assess the baseline method and the 3 longitudinal methods (Table 3). The 3 longitudinal methods had at least 10.5% higher power than the baseline method. Among the longitudinal methods, the power of both mean measure and 2-stage methods was comparable (41% and 40.5%, respectively) and substantially higher than that of the linear mixed-effects (LME) method (32.5%). None of the 4 methods showed elevated type I error because the genomic control value ranged from about 0.98 to 1.034.

**Table 3:** Power calculation of all 4 methods (based on the 200 simulations)

Method	Baseline	Mean measure	Two-stage	LME
Power	22%	41%	40.5%	32.5%

## Discussion and conclusions

For both traits, the genes identified by the 3 longitudinal methods were consistent, but different from those found with the baseline approach. From the perspective of computational time, the mean measure and 2-stage methods were more computer efficient than the LME method. Furthermore, these 2 longitudinal methods were more powerful than the LME method. These 2 methods can act as efficient and powerful "substitutes" for LME. The mean measure method worked as well as the 2-stage method, identifying the same genes. The signals found with the 2-stage method (third row of Manhattan plot in Figure 1) were almost identical to those with the LME method, for both SBP and DBP. Therefore, we concluded that the mean measure and 2-stage methods were 2 efficient ways to analyze longitudinal data when the goal is to examine level of a trait. Only the longitudinal approach can evaluate associations with trends over time.

## Competing interests

The authors declare that they have no competing interests.

## Authors' contributions

SW, WG, JN, CA, CTL, LAC designed the overall study, SW, WG, JN, CA conducted statistical analyses and SW, WG, JN drafted the manuscript. All authors read and approved the final manuscript.
